# Neurorehabilitation: Neural Plasticity and Functional Recovery

**DOI:** 10.1155/2017/3764564

**Published:** 2017-04-20

**Authors:** Toshiyuki Fujiwara, Nam-Jong Paik, Thomas Platz

**Affiliations:** ^1^Department of Rehabilitation Medicine, Juntendo University Graduate School of Medicine, 2-1-1 Hongo, Bunkyo, Tokyo 113-8421, Japan; ^2^Department of Rehabilitation Medicine, Seoul National University College of Medicine, Seoul National University Bundang Hospital, 173-82 Gumi-Ro, Bundang-Gu, Seongnam 13620, Republic of Korea; ^3^BDH-Klinik Greifswald, Centre for Neurorehabilitation, Intensive Care, and Spinal Cord Injury Unit, Universität Greifswald, Karl-Liebknecht-Ring 26a, 17491 Greifswald, Germany

Neurorehabilitation plays an important role for neural plasticity and functional recovery following neurological disease. Neurorehabilitation is based on rehabilitation medicine, neuroscience, and neurophysiology. This special issue focused on the efficacy and mechanism by which neurorehabilitation can induce neural plasticity and functional recovery.

Articles published in this special issue covered neurorehabilitation following stroke, spinal cord injury, and other neurological disorders.

T. Fujiwara et al. reviewed the neurorehabilitation using electromyography- (EMG-) controlled neuromuscular electrical stimulation for upper extremity motor function following stroke. This review showed that application of wearable EMG-controlled NMES for 8 hours in daytime improved both arm and hand function and can induce plastic change in intracortical interneuron and spinal reciprocal interneuron.

J. Fu et al. reviewed the functional recovery induced by the exercise after spinal cord injury. Therapeutic exercise can induce reshaping of the skeletal muscle, physiological change of spinal motor neuron, and remodeling of the motor cortex.

Neurophysiology and neuroimaging are great tools for revealing neural plasticity induced by neurorehabilitation.

Neuroimaging studies in this special issue revealed novel findings of cortical reorganization following spinal cord injury, facial nerve palsy, hearing loss, and aerobic exercise in older adults.

Neurophysiological studies in this special issue revealed neural activity related to reduction of gait speed in Parkinson's disease and functional recovery of hemiplegia following stroke.

Advanced neurophysiological and neuroimaging techniques provided new insight into the functional recovery in neurological disorders.

We hope this special issue provides further knowledge of neurorehabilitation.

*Toshiyuki Fujiwara* *Nam-Jong Paik* *Thomas Platz*

